# Short-Term Reactions to Oliguria in Critically Ill Patients: A Retrospective Cohort Study

**DOI:** 10.3390/jcm14093107

**Published:** 2025-04-30

**Authors:** Dekel Stavi, Amir Gal Oz, Nimrod Adi, Asaph Nini, Yoel Angel, Andrey Nevo, Daniel Aviram, Itay Moshkovits, Yael Lichter, Ron Wald, Noam Goder

**Affiliations:** 1 Division of Anaesthesia, Pain Management and Intensive Care, Tel Aviv Sourasky Medical Center, Weizmann 6, Tel Aviv 6423919, Israel; dekel.stavi@gmail.com (D.S.); amirgo@tlvmc.gov.il (A.G.O.); yoela@tlvmc.gov.il (Y.A.); aviramdaniel@gmail.com (D.A.); itaymoshko@gmail.com (I.M.); 2 Faculty of Medicine, Tel Aviv University, Tel Aviv 69978, Israel; 3 Perioperative Medicine, Critical Care Department, University College London Hospital NHS Foundation Trust, 235 Euston Rd, London NW1 2BU, UK; 4 Critical Care Department, University College London Hospital NHS Foundation Trust, 235 Euston Rd, London NW1 2BU, UK; drylichter@gmail.com; 5 Division of Nephrology, St. Michael’s Hospital, 36 Queen St, Toronto, ON M5B 1W8, Canada; 6 Li Ka Shing Knowledge Institute of St. Michael’s Hospital, 209 Victoria St, Toronto, ON M5B 1T8, Canada; 7 Division of Surgery, Tel Aviv Sourasky Medical Center, Weizmann 6, Tel Aviv 6423919, Israel

**Keywords:** oliguria, critical care, urinary output, fluid balance

## Abstract

**Background/Objective**: Oliguria is common in critically ill patients and may indicate impaired kidney perfusion or acute injury, contributing to increased mortality. Effective management is essential to improve outcomes. To assess clinician reactions to oliguria and evaluate the effectiveness of fluid bolus and furosemide interventions. **Methods**: A retrospective cohort study was conducted using ICU data from a single center (2017–2023). Oliguria was defined as two consecutive hours of urine output < 20 mL/h following at least three hours > 20 mL/h. Clinicians’ reactions within four hours were categorized as no intervention, fluid bolus (>250 mL), or furosemide administration. Outcomes included urine output, fluid balance, and serum creatinine. **Results**: Among 4987 oliguria episodes, 4007 events in 1825 patients were analyzed: no reaction (2536), fluid bolus (923), and furosemide (548). Furosemide significantly increased urine output (53.9 to 75.3 mL/h, *p* < 0.001), while fluid bolus had no significant effect. Resolution of oliguria (mean urine output > 40 mL/h for 5 h post-intervention) was more frequent with furosemide (66.4%) than with fluid bolus (28.4%) or no reaction (27.6%) (*p* < 0.001). Treatment choices varied significantly among ICU attendings (*p* < 0.001). **Conclusions**: Furosemide was more effective than fluid bolus or no treatment in improving urine output and resolving oliguria. The observed variation in clinician practices underscores the need for standardized management protocols to enhance patient care.

## 1. Introduction

Oliguria, commonly encountered among critically ill patients, serves as a crucial marker of underlying physiological disturbances [1]. According to KDIGO guidelines, oliguria is defined as a urine output reduction below 0.5 mL/kg/h for six hours or more [2]. Traditionally, oliguria has been characterized by urine output less than 400 mL/day, equivalent to approximately 15–20 mL/h or 0.2–0.3 mL/kg/h, and is associated with significant clinical outcomes, including increased morbidity, mortality, and acute kidney injury (AKI) [3,4]. Recent studies have further indicated that shorter episodes of oliguria, specifically durations of 3–6 h at thresholds around 0.2 mL/kg/h, may more accurately predict critical clinical outcomes such as mortality and AKI progression [5,6].

Acute kidney injury (AKI) is defined by parameters of urine output and serum creatinine (sCr) and is associated with increased mortality in the ICU [2,7]. While some episodes of oliguria do not meet AKI criteria by creatinine measurements, and the clinical significance of isolated transient oliguria remains uncertain, other studies report similar ICU mortality in oliguric patients with or without sCr increase [8,9]. Nonetheless, whereas increments in sCr take time to develop, urine output may serve as a real-time indicator of kidney perfusion and function, enabling physicians to intervene and potentially alter the course of worsening kidney injury due to hypovolemia or other adverse effects of fluid overload [8,10,11].

When confronted with oliguria in the intensive care setting, clinicians must consider various clinical factors concerning the underlying disease and hemodynamic and volume status. Specifically, they must assess whether the decrease in urine output reflects an appropriate consequence to hypovolemia while glomerular and tubular function remain intact or if it signifies acute tubular necrosis [12]. The challenge lies in differentiating between these mechanisms and selecting the appropriate reaction, as an incorrect intervention may exacerbate kidney function deterioration or lead to increased volume overload, which also carries a potential risk of harm [13]. Despite a wide variety of clinical tools to assess hemodynamic as well as fluid responsiveness, a physician’s reaction is often based upon clinical understanding and judgment.

This study aims to elucidate the clinician reactions to oliguria episodes in critically ill patients, whether it is to observe only, use diuretics or give a fluid bolus. Furthermore, we will aim to shed light on the course of oliguria and outcomes with common therapeutic interventions, such as fluid and diuretic administration.

## 2. Methods

### 2.1. Ethics

Ethical approval for this study (IRB No. TLV-0061-24) was obtained from the Sourasky Medical Center Ethics Committee, 6 Weizmann Street, Tel Aviv, Israel, on 15 February 2024. The requirement for informed consent was waived as all data were fully anonymized prior to access and analysis. No AI technologies were used in the design, analysis, or writing of this study.

### 2.2. Study Design and Data Source

This retrospective cohort study analyzed data extracted from the Tel Aviv Sourasky Medical Center (TASMC) General ICU database (MetaVision, IMDsoft) between 2017–2023. In this study, oliguria was defined as two consecutive hours of urine output less than 20 mL/h following at least three hours with urine output greater than 20 mL/h. We selected this lower threshold to identify oliguria episodes more sensitively. Furthermore, the threshold we chose aligns closely with our clinical practice, as oliguria episodes are usually reported by nursing staff when urine output decreases to approximately 20 mL/h over two or more hours. Urine output was measured routinely every hour using a closed urinary collection system with a graduated urometer, allowing accurate measurement in increments as small as 5 mL. Physician reactions to oliguria were classified into three categories: no reaction (observation without intervention), administration of a fluid bolus (>250 mL) [14,15,16], and administration of furosemide, all within four hours from documented oliguria.

The patient-level data included age, sex, and pre-existing conditions (hypertension, diabetes mellitus, heart failure, coronary artery disease, liver disease, metastatic cancer, hematologic malignancy, and HIV/AIDS status); admission category (surgical, medical, major trauma, or sepsis); ICU length of stay (LOS), ICU mortality (deceased), and body weight. Additionally, for each oliguria episode, we recorded the sequential organ failure assessment (SOFA) score on the relevant day; average hourly UO four hours before the oliguria episode; average hourly urine output five hours after the oliguria episode; urine output during the 24 h period preceding the oliguria episode; 24 h urine output on the day of the oliguria episode; fluid balance on the days before and after oliguria; previous day cumulative fluid balance; hourly mean arterial pressure (MAP), calculated as an average from minute-by-minute data at the hour of oliguria; support with norepinephrine at any time during the hour of recorded oliguria; mechanical ventilation; serum creatinine, and lactate, sodium, and hemoglobin concentration. 

### 2.3. Statistical Analysis

Continuous variables are presented as means and standard deviations (SD) or medians with interquartile ranges (IQR), as appropriate. Categorical variables are reported as counts and percentages of participants within each group. Normally distributed variables were compared with one-way ANOVA. Variables that were not normally distributed were compared with nonparametric tests. Categorical variables were compared with the chi-square test. All oliguria events in the same patient were included. In addition, a patient with multiple oliguria events could contribute to different “reaction categories”. In this study, success in treating oliguria was defined using two criteria. The primary definition of success was an average urine output exceeding 40 mL/h for five hours following the oliguria recording. The secondary exploratory definition of success was a tripling of the average urine output over five hours compared to the lowest average urine output during the two-hour period of severe oliguria. To explore the predictors of achieving an average urine output greater than 40 mL/h following intervention for oliguria, a logistic regression analysis was utilized on the first oliguria episode for each patient. The model included variables such as patient gender, age, weight, SOFA score on the day of oliguria, use of norepinephrine at the hour of oliguria, mean arterial pressure (MAP), mechanical ventilation status, serum creatinine level, lactate level, and the type of physician reaction to oliguria.

To analyze repeated measurements within the same patient, we employed a linear mixed-effects model. This statistical approach explicitly accounted for patient-level clustering and repeated episodes within patients by specifying episodes nested within subjects. The model included fixed effects for physician reaction, timepoint (before vs. after intervention), and their interaction. This analysis allowed us to evaluate both within-subject changes (differences over time within individuals) and between-subject differences (differences between intervention groups). We reported estimated marginal means and their 95% confidence intervals. Pairwise comparisons were adjusted for multiple testing using the Bonferroni correction. Analyses were performed using IBM SPSS version 29. 

## 3. Results

We identified 4987 oliguria episodes. After excluding 70 cases where both furosemide and intravenous fluids were administered for the same oliguria episode and 910 events lacking data on average urine and creatinine levels before and after intervention, the analytical cohort comprised 4007 episodes involving 1825 patients.

Physician reactions to oliguria episodes were as follows: no reaction (2536 events); fluid bolus (923 events); and furosemide bolus (548 events). Since a single patient may have been subjected to different reactions across multiple oliguria episodes, we characterized individual patients based on data from their first oliguria event and the physician reaction that ensued.

Table 1 displays differences in baseline characteristics among patients, categorized by the physicians’ reaction to the initial oliguria event. A background of hypertension was most common in patients who received furosemide in reaction to oliguria. A surgical diagnosis was most common among those receiving a fluid bolus, whereas a medical diagnosis was most common among those receiving furosemide.

When analyzing differences among groups based on physicians’ reactions to oliguria, as illustrated in Table 2, episodes treated with furosemide had several notable features. Firstly, the episodes were associated with significantly lower SOFA scores (5.8 ± 3.1 compared to 6.4 ± 3.6 and 6.3 ± 3.7 in the no reaction and fluid groups, respectively, *p* < 0.001). Moreover, episodes in which the reaction was a furosemide bolus occurred at a later stage of hospitalization, with a median of 4.7 days since ICU admission (IQR 2.2, 10.6) compared to 3 (IQR 1.2, 7.7) and 1.7 (IQR 0.6, 5.4) days in the no reaction and fluid groups, respectively (*p*-value < 0.001). There were no significant differences in the percentages of oliguric patients experiencing hemodynamic instability requiring noradrenaline support between the no reaction group (30.3%) and the fluid group (33.5%). However, the furosemide group had a significantly lower incidence of noradrenaline support at 19.5% (*p* < 0.001).

Additionally, Table 2 shows that daily urine output and fluid balance varied significantly based on physician reactions. The average urine output was highest in the furosemide group at 1584 ± 849 mL/d, compared to 1014 ± 645 mL/d in the no reaction group and 978 ± 583 mL/d in the fluid group (*p* < 0.001). For fluid balance, the no reaction group averaged 941 ± 1411 mL/d, the fluid group 1914 ± 1661 mL/d, and the furosemide group 57 ± 1305 mL/d, with significant differences across all groups (*p* < 0.001).

### 3.1. Average Urine Output and Serum Creatinine Before and After Oliguria Diagnosis According to Physicians’ Reaction

**Figure 1:**
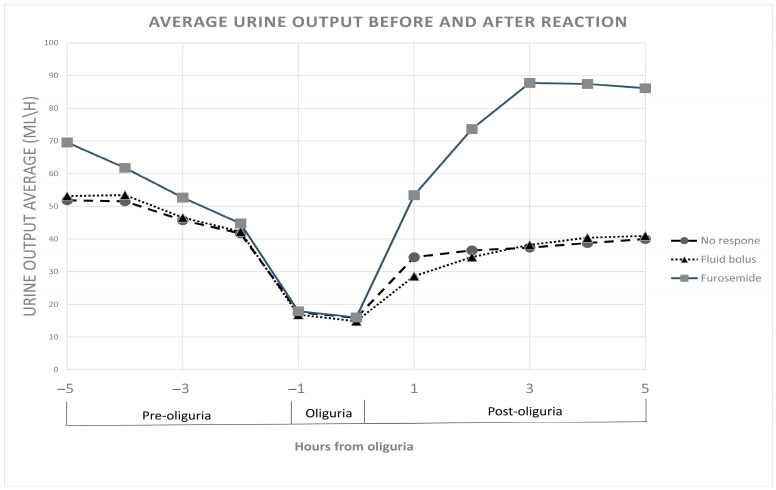
Average urine output before and after reaction. Average urine output in the four hours prior to oliguria detection and the change in urine output following physicians’ reaction.

In the four hours before oliguria was recorded, the no reaction group had an average urine output of 46.7 mL/h (95% CI: 45.4 to 48.1 mL/h), which decreased significantly to 37.5 mL/h (95% CI: 36.0 to 39.0 mL/h, *p* < 0.001) in the 5 h following the oliguria episode. In the fluid bolus group, the starting average urine output was 45.5 mL/h (95% CI: 43.5 to 47.5 mL/h), and this decreased significantly to 36.2 mL/h (95% CI: 33.8 to 38.5 mL/h, *p* < 0.001) following the intervention. The furosemide bolus group exhibited a significant increase from an initial average urine output of 53.9 mL/h (95% CI: 51.2 to 56.5 mL/h) to 75.3 mL/h (95% CI: 72.2 to 78.3 mL/h, *p* < 0.001) after treatment (Figure 1). Following the oliguria episode, there was no significant difference between the fluid bolus group and the no reaction group (*p* = 0.973), while both groups were significantly different from the higher output in the furosemide group (both *p* < 0.001).

In the investigation examining serum creatinine (sCr) levels in relation to physician reaction to oliguria, pre-reaction morning blood tests showed that the no reaction group had a median sCr level of 1.44 mg/dL (95% CI: 1.38–1.50). In comparison, the furosemide bolus group had a median of 1.38 mg/dL (95% CI: 1.30–1.46), and the fluid bolus group had a median of 1.35 mg/dL (95% CI: 1.28–1.42), with significant differences between groups (*p* = 0.004).

The morning following the oliguria episode, there was a significant overall increase in sCr (*p* = 0.001). Specifically, the no reaction group had a mean rise in sCr of 0.02 mg/dL (95% CI: −0.01–0.05, *p* = 0.285). The fluid bolus group had a mean rise of 0.06 mg/dL (95% CI: 0.01–0.11, *p* = 0.019), and the furosemide bolus group had a mean rise of 0.07 mg/dL (95% CI: 0.00–0.13, *p* = 0.044). There was no significant difference between groups in the magnitude of creatinine change (*p* = 0.192).

### 3.2. Comparative Analysis of Success Criteria Across Treatment Reactions

Successful resolution of oliguria (average urine output > 40 mL/h for 5 h following the oliguria event) was most likely following receipt of a furosemide bolus (success rate 66.4% vs. 28.4% for those who received a fluid bolus vs. 27.6% for those with no intervention, *p* < 0.001). In a multivariable analysis, receipt of furosemide was strongly associated with the successful resolution of oliguria (OR 5.315, 95% CI: 3.310–8.537). The following factors were significantly associated with a lower success rate in resolving oliguria: age (OR = 0.988 per year, 95% CI: 0.980–0.996), use of norepinephrine at the hour of oliguria (OR = 0.686, 95% CI: 0.477–0.985), and serum creatinine level (OR = 0.605 per 1 mg/dL rise, 95% CI: 0.506–0.723).

The secondary success criteria, defined as a tripling of urine output from the recorded oliguria, also showed significant differences among the intervention groups. Success rates were 19.7% for no intervention, 24.9% for fluid bolus, and 57.7% for the furosemide bolus. The differences between all the groups were statistically significant (*p* < 0.001).

### 3.3. Assessing the Impact of Physician Level of Training on Oliguria Treatment Decisions and Variability Among ICU Physicians

**Figure 2:**
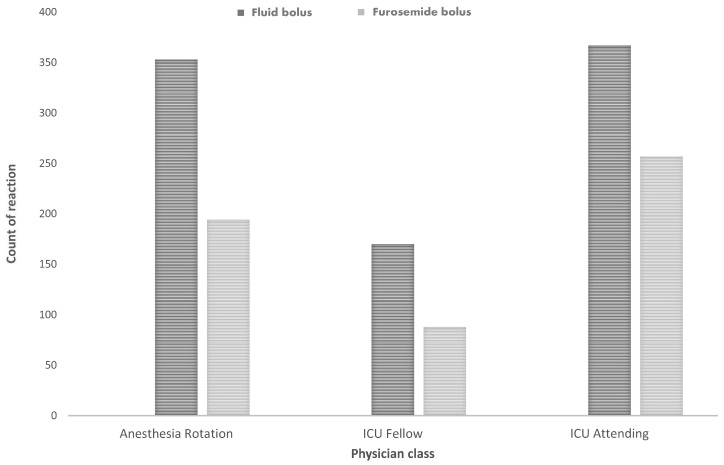
Reaction to oliguria by physicians’ level of training. Reactions to oliguria treatment with fluid or furosemide infusion among physicians at various training levels.

To further assess the reasoning behind each treatment choice, an analysis was conducted to examine the influence of physician training levels on oliguria management and the variability in treatment selections among ICU attendings. A total of 1429 cases were evaluated, each attributed to a specific physician (cases of no reaction could not be attributed to any physician), aiming to elucidate the impact of physician training on the decision to administer either a fluid bolus or furosemide in reaction to oliguria (Figure 2).

The distribution of treatment choices among different physician categories, along with corresponding case counts, was as follows: Anesthesia rotation physicians (n = 547) opted for a fluid bolus in 64.5% of cases and a furosemide bolus in 35.5%; ICU fellows (n = 258) chose a fluid bolus in 65.9% of instances and a furosemide bolus in 34.1%; ICU attending physicians (n = 624) selected a fluid bolus for 58.8% of episodes and a furosemide bolus in 41.2% of cases (*p* = 0.055).

**Figure 3:**
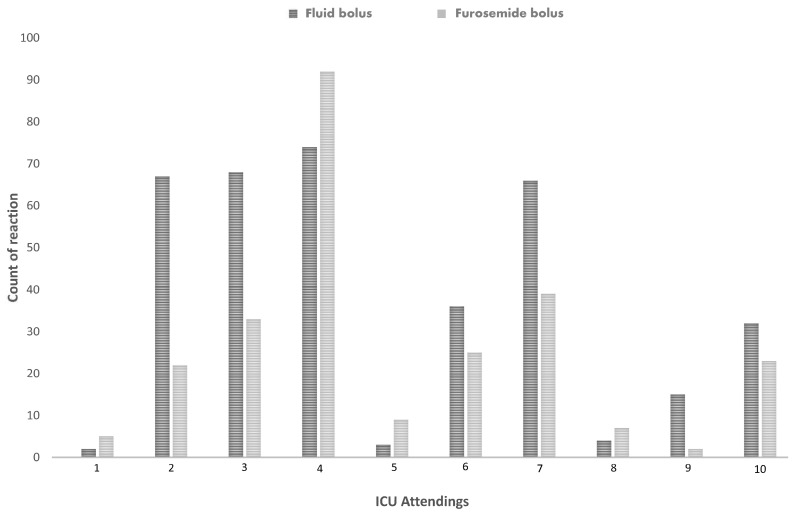
Specific ICU attendings reaction to oliguria. ICU attendings different reactions to oliguria either by fluid or furosemide infusion.

Upon analyzing the variability among specific ICU attendings, significant differences in treatment preferences were noted. Some attendings exhibited a marked preference for furosemide bolus over fluid bolus, while others significantly favored the fluid bolus. The observed variability in preferences among ICU attendings was statistically significant, with *p* < 0.001 (Figure 3).

### 3.4. Impact of Edema Presence on Physician Reaction to Oliguria Management

Edema presence or absence was noted in the medical records prior to 70% of oliguria events. Among events without edema, no reaction was recorded in 61.9% (n = 1355), 25.2% (n = 522) received fluids, and 12.9% (n = 283) were treated with furosemide. Conversely, among patients with edema, 65.5% (n = 395) had no reaction recorded, 16.6% (n = 100) received fluids, and 17.9% (n = 108) were administered furosemide. These differences were statistically significant (*p* < 0.001). Initial urine output was comparable across treatment groups in both edema and non-edema groups. Post-treatment, the furosemide group exhibited a significant increase in urine output compared to those with no reaction or those who received fluid boluses, regardless of edema presence, consistent with findings from the overall study population described earlier (*p* < 0.001).

## 4. Discussion

Our single-center study retrospectively assessed ICU physicians’ reactions to oliguria. We reviewed our ICU database for new oliguria episodes, the reactions undertaken, and outcomes in terms of urinary output and fluid balance. Given the variability in oliguria management, we also evaluated the consistency among physicians.

Several key findings emerged from our observations:

(1) Clinical characteristics, including lower SOFA scores and advanced ICU days since admission, were distinctive among groups receiving different interventions, particularly in determining the administration of furosemide. (2) Previous assessments of urinary output and fluid balance potentially influenced physicians’ reactions to oliguria. (3) Furosemide usage was lower in patients supported by noradrenaline. (4) Fluid boluses did not significantly affect urine output, showing no difference from the no reaction group. In contrast, furosemide significantly elevated urine output. (5) Fluid balance varied significantly between groups, with those receiving fluids having the highest balance, followed by the no reaction group, and the furosemide group exhibiting the lowest balance. (6) When considering reactions only, physicians generally preferred administering fluid boluses over a furosemide push, with considerable variability among ICU attendings. (7) A furosemide bolus was more commonly administered when edema was observed. However, the same pattern of reaction to furosemide, fluids, or no reaction was noted in both edematous and non-edematous patients.

Oliguria commonly occurs in critically ill patients, and pinpointing its exact cause can be challenging due to various underlying mechanisms. It is often uncertain whether the sudden decrease in urinary output is a result of evolving kidney failure or an appropriate reaction to hypovolemia. While AKI could be diagnosed by both parameters of urine output and serum creatinine, the second is a later observation, and thus, clinicians confronted with oliguria are driven to initiate an investigation aiming to diagnose the etiology and to decide about the appropriate management.

Clinicians often view urinary output as indicative of internal organ perfusion, but treatment needs to be carefully tailored to the clinical situation. Many conditions causing reduced urinary output may not respond to fluid administration, and in some cases, administering fluids may even be detrimental despite hemodynamic instability [2]. By the same token, diuretics are appropriate for patients who are volume expanded but can be harmful if there is actual volume contraction [1].

Physiologically, excluding mechanical post-renal causes, factors such as antidiuretic hormone secretion, activation of the sympathetic nervous system and the renin-angiotensin system due to hypovolemia, or decreased glomerular and tubular function can lead to a sudden decrease in urinary output [9]. Clinically, multiple factors are taken into consideration when evaluating oliguria, including the patient’s primary reason for ICU admission, current hemodynamic status, intravascular and extravascular volume status, and medications [1]. Processing this information and distinguishing between various causes is crucial as it necessitates different clinical interventions. However, identifying the specific cause can be challenging, leading many clinicians to rely on their clinical judgment to decide whether to administer fluids or diuretics.

A diverse approach to oliguria, depending on the SOFA score, ICU admission day, and overall fluid balance, may signify a more tailored reaction to the specific patient’s condition. As patients progress in their ICU stay with lower SOFA scores, a shift towards de-escalation and de-resuscitation becomes necessary [13].

In instances of newly occurring oliguria, fluid infusions in our study typically did not enhance urinary output and were associated instead with a further inflation of the cumulative fluid balance. Conversely, furosemide infusion often led to heightened urinary output and was associated with reduced fluid balance. This observation warrants discussion within the context of the patient’s ICU care stage when oliguria emerged, distinguishing earlier stages where intravascular hypovolemia due to various factors like trauma, bleeding, septic shock, or inadequate fluid intake may be prevalent from later ICU stages.

The reaction to oliguria when the patient is unstable and requiring noradrenaline support should also consider the underlying etiology and complex hemodynamic. Hemodynamic instability does not necessarily indicate intravascular depletion and fluid responsiveness, while being fluid responsive does not always imply that fluid infusion is clinically appropriate, considering other organ perspectives such as extravascular fluid accumulation.

The disparity in attending physicians’ approaches to oliguria underscores the complexity and multifactorial nature of such decisions across a broad spectrum of considerations. A more structured approach to acute oliguria could potentially enhance overall consistency and management in such scenarios.

Several limitations should be considered when interpreting the findings of this study. First, this is a retrospective, single-center analysis, potentially limiting generalizability, as management practices and patient populations can vary significantly between institutions. Second, although the large and heterogeneous cohort of critically ill patients provides a broad and realistic perspective, it simultaneously limits our ability to perform fully individualized patient assessments. Third, the study period (2017–2023) covers several years, including the COVID-19 pandemic, which might have influenced clinical decision-making and introduced temporal variability in practice. Fourth, due to the retrospective nature of the study, we could not systematically capture detailed hemodynamic evaluations or assessments of intravascular volume status (e.g., point-of-care ultrasound such as inferior vena cava diameter, minimally invasive monitoring), thus limiting our ability to interpret the appropriateness of clinician reactions based on objective volume status indicators. Fifth, although we recorded general inotrope use, we could not specifically determine which patients received inotropes for cardiac insufficiency, which might introduce an unmeasured bias. Sixth, a substantial number of oliguria episodes did not trigger an immediate documented clinical reaction within the predefined 4-hour observation window, potentially indicating passive management strategies or delayed reactions not captured by our analysis. Seventh, our study used increased urine output as a surrogate marker of treatment success, which, while practical, does not necessarily equate directly to renal recovery or improvement in kidney function. Finally, we did not evaluate longer-term clinical outcomes such as morbidity, mortality, or persistent kidney injury, which are important but beyond the scope of this study. These acknowledged limitations highlight the need for prospective, controlled studies incorporating detailed bedside hemodynamic and volume status evaluations to validate our findings further and inform clinical guidelines.

## 5. Conclusions

There is significant variability among physicians in their approaches to newly onset oliguria in the ICU, highlighting the need for a more rational and structured management approach. Our primary aim in this study was to characterize how frequently clinicians reacted to oliguria episodes, identify factors influencing therapeutic choices (such as physician seniority), and assess how these reactions were associated with immediate clinical outcomes—specifically urine output and serum creatinine levels. We observed that fluid bolus administration was primarily associated with increased fluid balance without a corresponding improvement in urine output, whereas furosemide administration was consistently associated with increased urine output. Rather than drawing broadly generalizable conclusions, these findings illustrate potential associations between clinical reactions and immediate outcomes, supporting the need to develop structured guidelines to optimize oliguria management in critically ill ICU patients.

## Figures and Tables

**Table 1:** Patients’ characteristics across reaction groups, categorized by reaction to the first oliguria episode in each patient.

Patients’ Characteristics n = 1825	No Reaction n = 1121		Furosemide Administration n = 177		Fluid Bolus n = 527		*p* Value
	Mean/Median	SD/IQR/%	Mean/Median	SD/IQR/%	Mean/Median	SD/IQR/%	
Age (years)	61.73	17.41	62.63	17.21	60.54	17.60	0.280
Sex (Female)	463 (a)	41.2%	90	50.8%	212 (a)	40.2%	0.036
ICU LOS (days)	6.65 (a)	[3.6, 12.9]	7.3	[4.2, 14.4]	6 (a)	[3.2, 11]	0.004
Deceased	152	13.6%	25	14.1%	72	13.7%	0.979
weight (kg)	78.6	24.0	79.7	26.3	76.4	19.9	0.247
**pre-existing conditions**							
Hypertension	380	33.9%	76	42.9%	152	28.8%	0.002
Diabetes mellitus	223	19.9%	46	26.0%	98	18.6%	0.101
Heart failure	65	5.8%	13	7.3%	24	4.6%	0.333
Coronary artery disease	178	15.9%	35	19.8%	78	14.8%	0.293
Liver disease	44	3.9%	4	2.3%	25	4.7%	0.338
Metastatic cancer	19	1.7%	5	2.8%	9	1.7%	0.565
Hematologic malignancy	22	2.0%	4	2.3%	9	1.7%	0.884
HIV/AIDS	10	0.9%	0	0.0%	5	0.9%	0.441
**Admission category—no. (%)**							
Surgery	194 (b)	17.4%	29	16.5%	120 (b)	22.8%	0.023
Medical	852 (a)	76.2%	137 (a)	77.8%	367	69.6%	0.010
Major trauma	75	6.7%	10	5.7%	42	8.0%	0.502
Sepsis	302 (a)	27.0%	54 (a)	30.7%	117	22.2%	0.038

(a) no difference between these groups (*p* > 0.05) with Bonferroni correction. (b) Statistical difference was observed only between these groups (*p* < 0.05) with Bonferroni correction. ICU LOS—intensive care unit length of stay, HIV—human immunodeficiency virus, AIDS—acquired immunodeficiency syndrome.

**Table 2:** Baseline physiological data collected surrounding each oliguria episode.

Per Specific Reaction n (Total Episodes) = 4007	No Reaction n = 2536		Furosemide Administration n = 548		Fluid Administration n = 923		*p* Value
	Mean/Median	SD/IQR/%	Mean/Median	SD/IQR/%	Mean/Median	SD/IQR/%	
Days from admission	3.00	[1.2, 7.7]	4.70	[2.2, 10.6]	1.70	[0.6, 5.4]	<0.001
SOFA score	6.4 (a)	3.6	5.8	3.1	6.3 (a)	3.7	<0.001
Daily urine (mL/d, before reaction)	1135 (a)	813	1484	889	1131 (a)	795	<0.001
Daily urine (mL/d, after reaction)	1014 (a)	645	1584	849	978 (a)	583	<0.001
Daily balance (mL/d, before reaction)	755	1564	162	1461	926	1670	<0.001
Daily balance (mL/d, after reaction)	941	1411	57	1305	1914	1661	<0.001
Previous day accumulative fluid balance	3374	7539	1338	8474	4164	7450	<0.001
Mean arterial pressure—mmHg	76.9 (a)	18.0	77.4 (a)	19.2	73.2	18.4	<0.001
Noradrenaline support	769 (a)	30.3%	107	19.5%	309 (a)	33.5%	<0.001
Mechanical ventilation support	1743	68.7%	390	71.2%	651	70.5%	0.389
Serum creatinine day of reaction—mg/dL	1.05	[0.60, 2.09]	0.90 (a)	[0.54, 1.64]	0.96 (a)	[0.63, 1.63]	<0.001
Continuous renal replacement therapy (CRRT)	31	1.2%	11	1.2%	6	1.1%	0.969
Serum creatinine day after reaction—mg/dL	1.08 (b)	[0.58, 2.24]	0.935 (b)	[0.53, 1.76]	1.03	[0.61, 1.78]	0.003
Lactate before reaction (mmol\L)	1.21	[0.87, 1.72]	1.12	[0.83, 1.55]	1.38	[0.95, 2.01]	<0.001
Lactate after reaction (mmol\L)	1.16	[0.83, 1.64]	1.12	[0.79, 1.49]	1.37	[0.94, 2.00]	<0.001
Hemoglobin—g/dL	9.50	2.06	9.50	2.04	9.63	2.24	0.238
Serum Sodium (mEq\L)	141.59 (a)	6.00	141.38 (a)	5.31	140.81	6.07	0.003

(a) no difference between these groups (*p* > 0.05) with Bonferroni correction. (b) Statistical difference was observed only between these groups (*p* < 0.05) with Bonferroni correction. SOFA—sequential organ failure assessment.

## Data Availability

The datasets used and/or analyzed during the current study are available from the corresponding author on reasonable request.

## Abbreviations

ICU	intensive care unit
LOS	length of stay
HIV	human immunodeficiency virus
AIDS	acquired immunodeficiency syndrome.
SD	standard deviations
IQR	interquartile ranges
SOFA	sequential organ failure assessment
sCr	serum creatinine
UO	urinary output
MAP	mean arterial pressure
AKI	acute kidney injury
